# Low-dimensional approximation searching strategy for transfer entropy from non-uniform embedding

**DOI:** 10.1371/journal.pone.0194382

**Published:** 2018-03-16

**Authors:** Jian Zhang

**Affiliations:** School of Mathematical Sciences, Zhejiang University, Hangzhou, China; University of Adelaide, AUSTRALIA

## Abstract

Transfer entropy from non-uniform embedding is a popular tool for the inference of causal relationships among dynamical subsystems. In this study we present an approach that makes use of low-dimensional conditional mutual information quantities to decompose the original high-dimensional conditional mutual information in the searching procedure of non-uniform embedding for significant variables at different lags. We perform a series of simulation experiments to assess the sensitivity and specificity of our proposed method to demonstrate its advantage compared to previous algorithms. The results provide concrete evidence that low-dimensional approximations can help to improve the statistical accuracy of transfer entropy in multivariate causality analysis and yield a better performance over other methods. The proposed method is especially efficient as the data length grows.

## Introduction

In the study of neuroscience, directed information measure has been proven as a critically important tool in a wide range of application scenarios. Over recent years, a variety of time series analysis methods have been introduced to identify the existence and direction of the interactions in nervous system, and there are two most commonly used methods: Granger causality (GC), which is derived from the field of econometrics [1], and its information-theoretic analog, transfer entropy (TE) [2, 3]. Both of them are based on the simple idea that if the prediction of a time series (the effect) could be improved by incorporating the knowledge of past information of a second one (the cause), then the latter is considered to have a causal influence on the former. Moreover, it has been proved [4] that there is a close connection between Granger causality and transfer entropy: these two measures are equivalent for time series under the assumption of Gaussianity. This fact leads to a bridge between econometric and information-theoretic predictive approaches for the evaluation of directed couplings. The result has also been extended to the condition of non-Gaussian probability density distributions [5]. For computationally efficiency, the exact formulation of conditional mutual information (CMI) derived from GC-TE equivalence has been frequently employed in the analysis of time series with high linearity, such as for fMRI data [6], while transfer entropy, as a generally model-free approach which does not require any prior assumptions about the probability distribution, has been mostly adopted to nonlinear complex systems such as in the field of physiology [7–9] or climatology [10–12].

Although transfer entropy has several practical advantages, it still remains some critical drawbacks to overcome. For example, if the dimension of the embedding space that spanned by the variables is relatively large, the estimation of mutual information which is required for the computation of transfer entropy will inevitably suffer from the problem “curse of dimensionality” [10, 13, 14], make it difficult to uncover correctly the underlying causal structure of data. To tackle this problem, it is needed to improve the choice of parameters to get a well fitted model. One promising approach is the shrinking strategy which is based on properly reduction for the overall parameters to be estimated. Several paradigms focus on this objective have been proposed by previous researchers, in econometrics it is called “subset regression” [15–18], a method relies on sequential t-tests and model selection criteria, or by carrying out branch-and-bound strategy to cut subtrees [19], while in physics it is usually under the name “Non-uniform Embedding” [7, 13, 20–22], a method relies on information-theoretic measures and non-uniform state space reconstruction. All of these paradigms are aiming to achieve a parsimonious model by picking up the most informative lags of driver variables that has a significant impact to the target variable, while restricting the state space by reducing the dimensionality.

In this paper we propose a paradigm that makes use of the low-dimensional approximation technique for conditional mutual information, which was originally derived from the study of information-theoretic criterion for feature selection. We employ this method to deal with the problem of searching lagged variables in the computing process of transfer entropy from non-uniform embedding. In the implementation of traditional scheme of non-uniform embedding, the main procedure is to reconstruct a future point of the target variable in the subspace of the joint state space spanned by lagged driven variables. It derives from a sequential selection method which updates the embedding vector progressively, taking all relevant lagged variables into consideration at each step, stopping under certain termination criterion to drop all the vectors on irrelevant lags, and finally identifying the set of components that associated with the most information transfer to the target process. The traditional termination criterion applied by previous researchers [9, 13, 23] is based on a statistical significant test for the conditional mutual information between candidate variables. In this article a new method modified by low-dimensional approximation searching strategy will be introduced to improve the accuracy and efficiency of previous TE algorithms.

The rest of this paper is organized as follows. In the next section, we provide a brief review of transfer entropy from non-uniform embedding and low-dimensional approximation paradigm in feature selection, and then describe in detail the formulation of our approach. In section 3, we present a number of simulation experiments to demonstrate the effectiveness of the proposed algorithm compared to previous methods, In section 4 we test our approach in analyzing intracranial EEG recordings from an epileptic patient to show its practical applicability on real-world data, and the conclusion will be summarized in the final section.

## Materials and methods

### Transfer entropy from non-uniform embedding

We start by presenting the definition of transfer entropy from non-uniform embedding [13, 20, 23], also known as lag-specific transfer entropy [9, 11], which is a measure to estimate the directional coupling within a dynamical system by using the conditional mutual information for variable selection in the procedure of mixed embedding. Let us firstly consider an overall dynamical system composed of *M* interacting subsystems, and suppose that we are interested in evaluating the information flow from a driving subsystem *X*, to the destination subsystem *Y*, in the presence of the remaining subsystems which are described by the set of processes $Z={\left\{Z^{\left(k\right)}\right\}}_{k=1,\ldots ,M-2}$. Here we discuss our work under the assumption that the states of the system can be described as a multivariate stationary stochastic process, and denoted by *X*_*n*_, *Y*_*n*_, and $Z_{n}$ as the observations obtained by sampling the processes at present time *n*, and accordingly, $X_{n}^{-}=\left[X_{n-1},X_{n-2},\cdots \right],Y_{n}^{-}=\left[Y_{n-1},Y_{n-2},\cdots \right]$, and $Z_{n}^{-}=Z_{n-1}⊕Z_{n-2}⊕\cdots$ as the respective sets of variables describing the past of the processes. Then, the (multivariate) transfer entropy from process *X* to *Y* conditioned on Z, which quantifies the information provided by the past of *X* about the present state of *Y* that has not been contained in the past of *Y* or any other processes included in Z, could be defined in the form of conditional mutual information $I(Y_{n};X_{n}^{-}|Y_{n}^{-}⊕Z_{n}^{-})$, or equivalently as a difference of two conditional entropies:

$$\begin{array}{ccc} T_{X\to Y|Z} & = & I(Y_{n};X_{n}^{-}|Y_{n}^{-}⊕Z_{n}^{-})\\ & = & H\left(Y_{n}|Y_{n}^{-}⊕Z_{n}^{-}\right)-H\left(Y_{n}|X_{n}^{-}⊕Y_{n}^{-}⊕Z_{n}^{-}\right) \end{array}$$ (1)

where *H*(⋅) stands for Shannon entropy, and *I*(⋅) stands for mutual information.

The crucial step in the procedure for estimating transfer entropy $T_{X\to Y|Z}$ from non-uniform embedding, is to build a conditioning vector *V*_*n*_ by a sequential selection procedure from the collection of candidate vectors including in the past of *X*, *Y* and *Z* with a fixed searching depth up to a maximum truncation lag *L*. The candidate vectors of all relevant processes compose the set $\Omega =\{X_{n-1},\ldots ,X_{n-L},Y_{n-1},\ldots ,Y_{n-L},Z_{n-1},\ldots ,Z_{n-L}\}$, and the information transfer to the target can be quantified by means of the sum of all contributions at different time lags. The searching strategy for significant components in Ω can be described as follows:

1. Starting with an empty vector $V_{n}^{\left(0\right)}=⌀$

2. At each step *k* ≥ 1, perform a searching procedure to select the most informative vector on a specific lag of past variables, $W_{n}^{\left(k\right)}$, that contribute most significantly to the target variable *Y*_*n*_, from the available candidate vectors $\Omega \backslash V_{n}^{\left(k-1\right)}$ (the subtraction from Ω by $V_{n}^{\left(k-1\right)}$), and construct the optimal conditioning embedding vector by adding $W_{n}^{\left(k\right)}$ to the already chosen (*k* − 1) vectors, i.e. $V_{n}^{\left(k\right)}=V_{n}^{\left(k-1\right)}⊕W_{n}^{\left(k\right)}$. Each candidate vector $W_{n}^{\left(k\right)}$ will be tested by a criterion through computing the maximum CMI between $W_{n}^{\left(k\right)}$ and the target vector *Y*_*n*_ conditioned on the already chosen vector $V_{n}^{\left(k-1\right)}$:

$$\begin{array}{c} W_{n}^{\left(k\right)}=\underset{W_{n}\in \Omega \backslash V_{n}^{\left(k-1\right)}}{argmax}I\left(Y_{n};W_{n}|V_{n}^{\left(k-1\right)}\right) \end{array}$$ (2)

3. Terminate the loop of selection when there is no $W_{n}^{\left(k\right)}$ can fulfill the significance test. The significance test is designed in line with the statistical method from previous studies [13, 22, 23], in each searching loop 100 surrogate data are generated by a random shift procedure on the time points of causal driver and target variables for zero-setting the significant elements at specific lags, and the CMI value is then compared with the empirical distribution of surrogates according to the null hypothesis of absent causality from the driver to the target, to see if the value is larger than the (1 − *α*)% percentile of the ensemble of the randomized $I(Y_{n};W_{n}|V_{n}^{\left(k-1\right)})$, before stop searching and return by $V_{n}=V_{n}^{\left(k-1\right)}$ as the conditioning embedding vector, which is composed of $V_{n}=V_{n}^{X}⊕V_{n}^{Y}⊕V_{n}^{Z}$, where $V_{n}^{X}$, $V_{n}^{Y}$ and $V_{n}^{Z}$ denote the components of *V*_*n*_ belonging respectively to *X*, *Y*, and Z.

4. Calculate the transfer entropy as:

$$\begin{array}{c} T_{X\to Y|Z}=H\left(Y_{n}|V_{n}^{Y}⊕V_{n}^{Z}\right)-H\left(Y_{n}|V_{n}\right) \end{array}$$ (3)

### The motivation to use low-dimensional approximation for transfer entropy

The above construction procedure for embedding vector *V*_*n*_ can be seen as a filter method of feature selection technique that relies on the criterion of CMI to sort and extract the most significant vectors from a high-dimensional state space, in order to establish a subset of history vectors that maximize the amount of information for predicting future state of target variable, and minimize the redundancy within the set of selected variables. To achieve this purpose without any prior assumptions of sample distribution, one needs to directly calculate the value of CMI from the estimated distribution $\hat{p}\left(\cdot \right)$, and rank the candidate vectors by the CMI in each step to search the most informative vector sequentially.

$$\begin{array}{ccc} CMI & = & I\left(Y_{n};W_{n}|V_{n}^{\left(k-1\right)}\right)\approx \hat{I}\left(Y_{n};W_{n}|V_{n}^{\left(k-1\right)}\right)\\ & = & \frac{1}{N}\sum_{i=1}^{N}log\frac{\hat{p}\left(Y_{n}\left(i\right)W_{n}\left(i\right)|V_{n}^{\left(k-1\right)}\left(i\right)\right)}{\hat{p}\left(Y_{n}\left(i\right)|V_{n}^{\left(k-1\right)}\left(i\right)\right)\hat{p}\left(W_{n}\left(i\right)|V_{n}^{\left(k-1\right)}\left(i\right)\right)} \end{array}$$ (4)

Nevertheless, as the dimension of conditioning embedding vector *V*_*n*_ is increasing with an growing number of candidate vectors *W*_*n*_ are added, the estimation of probability distributions $\hat{p}\left(\cdot \right)$ from a higher dimensional *V*_*n*_ becomes less reliable, which unavoidably renders the criterion of CMI more problematic, and may lead to inaccurate judgements for the inclusion/exclusion of candidate vectors. At this point the major barrier of uniform embedding method of TE—the curse of dimensionality which we hope to bypass through the non-uniform embedding scheme—reemerges. This impediment derives from the sparsity of the available data of an increasing volume of state space that makes the estimation of entropy rates progressively decrease towards zero [14]. For the calculation of TE from multivariate time series this problem is always aggravated by the limited length of data, which may commonly happen in physiological systems as in the case of EEG analysis. To overcome this obstacle, in the following sections we will present a low-dimensional approximation paradigm for TE estimation and test its effectiveness.

### Low-dimensional approximation strategy for CMI

The problem of estimating high-dimensional CMI from small samples is a long-standing challenge in the field of information theoretic feature selection and several well-accepted techniques have existed in published literature, for instance, MIFS criterion [24], JMI criterion [25], CMIM criterion [26], MRMR criterion [27], CIFE criterion [28], DISR criterion [29], etc. In recent years Brown et al. [30] have shown that the above various feature selection heuristics are all approximate iterative maximisers of the conditional likelihood, which can be interpreted in a unifying framework of conditional likelihood maximisation under certain assumptions of independence. Hence all the methods can be rewritten within a parameterized general criterion:

$$\begin{array}{c} J\left(W_{k}\right)=I\left(W_{k};Y_{n}\right)-\beta \sum_{W_{j}\in V}I\left(W_{k};W_{j}\right)+\gamma \sum_{W_{j}\in V}I\left(W_{k};W_{j}|Y_{n}\right) \end{array}$$ (5)

and the differences among them are determined by the scaling factor *β*/*γ*, for example, the CIFE criterion can be obtained with *β* = *γ* = 1, the MRMR criterion can be obtained with *β* = 1/|*V*| and *γ* = 0, and the JMI criterion is under the case of *β* = *γ* = 1/|*V*|. In [31] the researchers keep on investigating the theoretical underpinnings of high dimensional CMI and provide a principled approach (RelaxMRMR) for modifying feature selection criterion in which takes into account higher order feature interactions by relaxing some assumption of conditional independence. Compared to (5), the main innovation of RelaxMRMR is on the redundancy term that incorporates the second-order interactions between the features, i.e., the three-way feature interaction terms *I*(*W*_*k*_;*W*_*i*_|*W*_*j*_).

$$\begin{array}{c} J\left(W_{k}\right)=I\left(W_{k};Y_{n}\right)-\beta \sum_{W_{j}\in V}I\left(W_{k};W_{j}\right)+\gamma \sum_{W_{j}\in V}I\left(W_{k};W_{j}|Y_{n}\right)-\delta \underset{W_{i},W_{j}\in V;i\neq j}{\sum \sum }I\left(W_{k};W_{i}|W_{j}\right) \end{array}$$ (6)

where *β* = *γ* = 1/|*V*| and *δ* = 1/|*V*| or 1/|*V*|(|*V*| − 1) (Form-1 and Form-2 in [31]).

In this paper, we investigate the above two low-dimensional approximation criteria as a substitute measure to the high dimensional CMI in (2), and compare the performance of the methods with the original non-uniform TE algorithm. Suppose the set of conditioning vectors we have already built is *V* = {*W*_1_, *W*_2_, …*W*_(*k*−1)_} and *Y*_*n*_ is the target variable. The formulations of low-dimensional approximations (LA) to CMI here we used are:

$$\begin{array}{c} \text{LA1:}\;W_{n}=\underset{W_{k}\in \Omega \backslash V}{argmax}\left\{I\left(W_{k};Y_{n}\right)-\frac{2}{\left|V\right|}\sum_{W_{j}\in V}I\left(W_{k};W_{j}\right)+\frac{2}{\left|V\right|}\sum_{W_{j}\in V}I\left(W_{k};W_{j}|Y_{n}\right)\right\} \end{array}$$ (7)

$$\begin{array}{ccc} \text{LA2:}\;W_{n} & = & \underset{W_{k}\in \Omega \backslash V}{argmax}\{I\left(W_{k};Y_{n}\right)-\frac{1}{\left|V\right|}\sum_{W_{j}\in V}I\left(W_{k};W_{j}\right)+\frac{1}{\left|V\right|}\sum_{W_{j}\in V}I\left(W_{k};W_{j}|Y_{n}\right)\\ & & -\frac{1}{\left|V|(|V|-1\right)}\underset{W_{i},W_{j}\in V;i\neq j}{\sum \sum }I\left(W_{k};W_{i}|W_{j}\right)\} \end{array}$$ (8)

In LA1 (7), we didn’t take into account the second-order interactions between the candidate vectors, but employed a larger factor *β* = *γ* = 2/|*V*| than the JMI criterion, in order to give an outweighed difference between the redundancy *I*(*W*_*k*_;*W*_*j*_) and conditional redundancy *I*(*W*_*k*_;*W*_*j*_|*Y*_*n*_), compared to the relevance term *I*(*W*_*k*_;*Y*_*n*_). In LA2 (8) we adopted the same formulation and parameters as in Form-2 in [31], in which the second-order interactions (three-way feature interaction terms) were considered with a normalization factor 1/|*V*|(|*V*| − 1).

### Estimator of entropy

In this paper, the entropy estimators adopted in the implementation of TE are consistent with previous studies in order to compare the algorithms on a fair basis. The first estimator we used is a binning estimator, which divides the observed state space into a set of equal partitions by a fixed number of quantization levels, and the probability distribution is estimated by relative frequencies of occurrence of the quantization values [32]. The second one is nearest neighbor (NN) estimator [33], it adapts the local neighborhood to the dimension of the state space and makes bias compensation in the estimation of entropies of variables in different dimension. The third one is a linear model-based estimator, which works under the assumption that the multivariate process has a joint Gaussian distribution. It has been demonstrated [4] that the conditional entropy terms for the TE under the Gaussian assumption can be quantified by means of linear regressions involving the relevant variables taken from the embedding vector.

All the above three entropy estimators are the same as the ones employed by previous researchers [9, 13, 14, 23], and in the following analysis these estimators are implemented by means of the respective functions from the MATLAB toolbox MuTE [23]. The primary aim of our proposed method is to avoid the high-dimensional CMI as the ranking criterion in the greedy search procedure for TE from non-uniform embedding, but to adopt the method of low-dimensional approximations to tackle the problem of dimensionality curse. From the simulations in the next section we will demonstrate that applying the low-dimensional approximation criteria as a substitute method for selecting the embedding vectors in TE calculation is beneficial for obtaining a better result with higher sensitivity and specificity under various circumstances.

## Simulation study

In this section, we present a series of simulation experiments for causality analysis to compare the performance of low-dimensional approximation methods described in the previous section with the traditional non-uniform TE methods, on a number of multivariate linear and nonlinear stochastic models with various coupling strengths at different interaction lags. In all simulations, 100 realizations were generated for each model with data length 256/512/1024 to assess statistically the sensitivity and specificity of the methods. And the threshold of significance test in the selection loop for candidate vectors was set as *α* = 0.05, the number of surrogates was fixed to 100.

In terms of sensitivity and specificity, we computed the TE results with respect to a varying length of the analysed data. And the ROC (Receiver Operating Characteristic) curves were obtained for all methods from 256 to 1024 time points. The amount of TP (ture positive), TN (true negative), FP (false positive) and FN (false negative) were estimated and classified by grouping all coupled directions (positives) and all uncoupled directions (negatives), to calculate the sensitivity = *TP*/(*TP* + *FN*), specificity = *TN*/(*TN* + *FP*), and F1 score = 2*TP*/(2*TP* + *FP* + *FN*).

For the first two linear models, we used the linear entropy estimator since it works optimally for the time series with a joint Gaussian distribution. Regarding the other three nonlinear models, binning and nearest neighbor estimators were employed to evaluate the difference of performance.

### Model A

**Fig 1:**
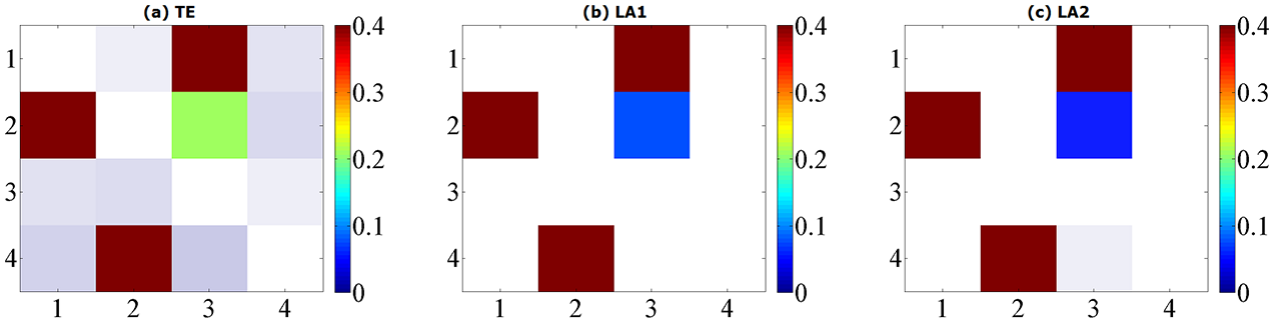
Matrix representations of the corresponding causalities for Model A. Retrieved by traditional TE method (a) and two low-dimensional approximation methods (b) (c) with linear estimator. The results are averaged over 100 simulations of 1024 time points and shown with color revealing the magnitude and transparency indicating the significance. The directions of causal influence are from row to column.

**Fig 2:**
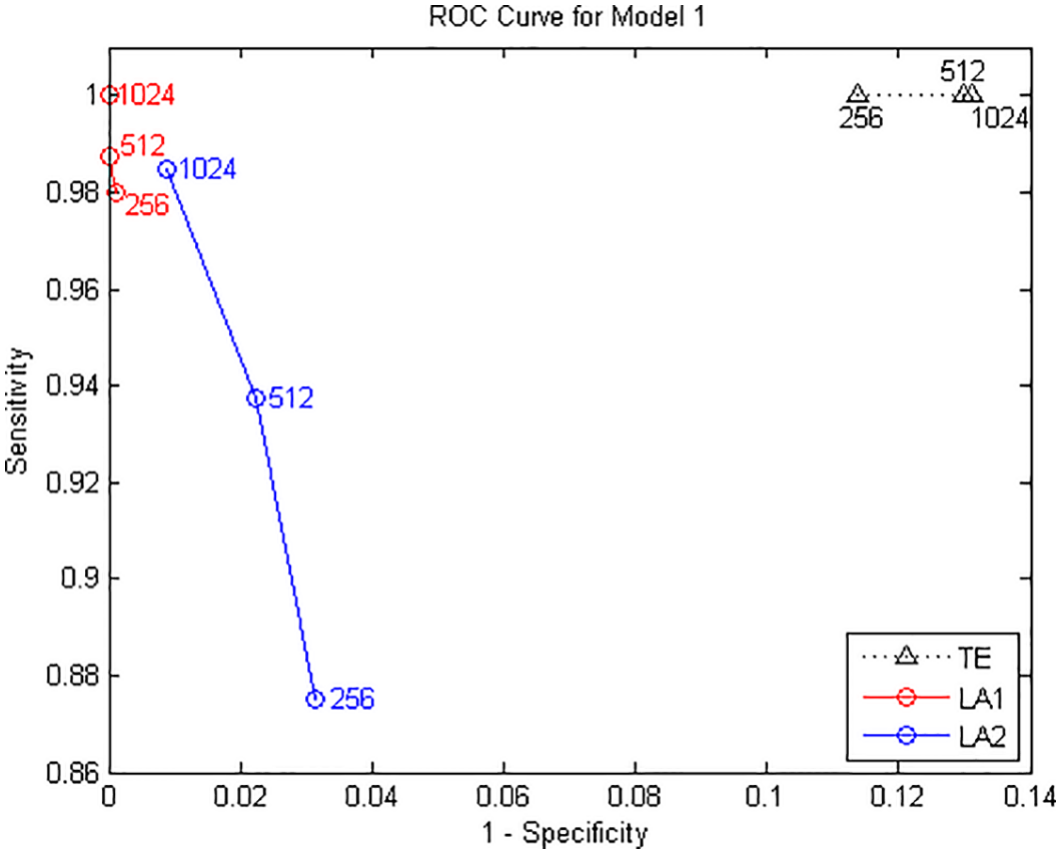
ROC curves for Model A. Sensitivity and specificity are obtained by gradually increasing the time series length from 256 to 1024 points.

Let us start with a linear vector autoregressive (VAR) model which is composed by 4 time series of order 5 (Model 1 in [34]). The equations for this model are:

$$\begin{array}{c} \left\{ \begin{array}{ccc} x_{1}\left(t\right) & = & 0.8x_{1}\left(t-1\right)+0.65x_{2}\left(t-4\right)+\epsilon_{1}\left(t\right)\\ x_{2}\left(t\right) & = & 0.6x_{2}\left(t-1\right)+0.6x_{4}\left(t-5\right)+\epsilon_{2}\left(t\right)\\ x_{3}\left(t\right) & = & 0.5x_{3}\left(t-3\right)-0.6x_{1}\left(t-1\right)+0.4x_{2}\left(t-4\right)+\epsilon_{3}\left(t\right)\\ x_{4}\left(t\right) & = & 1.2x_{4}\left(t-1\right)-0.7x_{4}\left(t-2\right)+\epsilon_{4}\left(t\right) \end{array}\right. \end{array}$$ (9)

where *ϵ*_*i*_(*t*), *i* = 1, …, 4 are unit Gaussian noise terms with zero mean and unit covariance matrix. We performed the causal analysis by setting the maximum lag according to the largest lag in the generating process, and evaluate the transfer entropy from non-uniform embedding by the traditional algorithm and our low-dimensional approximations searching methods (LA1, LA2). The results from model A for data length of 1024 points are shown in Fig 1, depicted by a causal matrix of interactions from row to column for each method, the ROC curves of 256/512/1024 data points are shown in Fig 2 and the values of sensitivity/specificity/F1 score listed in Table 1. The true causal connections in model A are at the matrix elements (1, 3), (2, 1), (2, 3), and (4, 2). For all three methods the deduced values of TE on these positions are always positive and high, demonstrate a good sensitivity for the true couplings. However we can notice that a number of false positives were given by the traditional method using the high dimensional CMI, especially for the large data of 1024 points. In contrast, although both low-dimensional approximation methods presented a relatively low rejection rate for the data of 256 and 512, for 1024 points they attained a better performance with respect to the sensitivity/specificity/F1 score. And LA1 in this model provides the best possible result compared to LA2.

**Table 1:** Sensitivity, specificity and F1 score values obtained from Model A.

	Sensitivity	Specificity	F1 score
256 points			
TE	1.000	0.886	0.898
LA1	0.980	0.999	0.989
LA2	0.875	0.969	0.903
512 points			
TE	1.000	0.870	0.885
LA1	0.988	1.000	0.994
LA2	0.938	0.978	0.946
1024 points			
TE	1.000	0.869	0.884
LA1	1.000	1.000	1.000
LA2	0.985	0.991	0.984

### Model B

The second model is another linear VAR of order 4 in five variables with unit Gaussian noise:

$$\begin{array}{c} \left\{ \begin{array}{ccc} x_{1}\left(t\right) & = & 0.4x_{1}\left(t-1\right)-0.5x_{1}\left(t-2\right)+0.4x_{5}\left(t-1\right)+\epsilon_{1}\left(t\right)\\ x_{2}\left(t\right) & = & 0.4x_{2}\left(t-1\right)-0.3x_{1}\left(t-4\right)+0.4x_{5}\left(t-2\right)+\epsilon_{2}\left(t\right)\\ x_{3}\left(t\right) & = & 0.5x_{3}\left(t-1\right)-0.7x_{3}\left(t-2\right)+\epsilon_{3}\left(t\right)\\ x_{4}\left(t\right) & = & 0.8x_{4}\left(t-3\right)+0.4x_{1}\left(t-2\right)+0.3x_{2}\left(t-2\right)+\epsilon_{4}\left(t\right)\\ x_{5}\left(t\right) & = & 0.7x_{5}\left(t-1\right)-0.5x_{5}\left(t-2\right)-0.4x_{4}\left(t-1\right)+\epsilon_{5}\left(t\right) \end{array}\right. \end{array}$$ (10)

**Fig 3:**
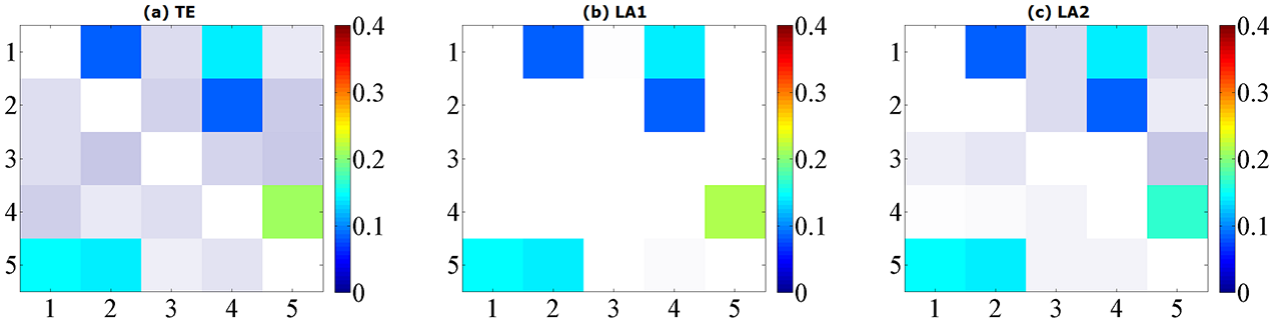
Matrix representations of the corresponding causalities for Model B. Retrieved by traditional TE method (a) and two low-dimensional approximation methods (b) (c) with linear estimator. The results are averaged over 100 simulations of 1024 time points and shown with color revealing the magnitude and transparency indicating the significance. The directions of causal influence are from row to column.

**Fig 4:**
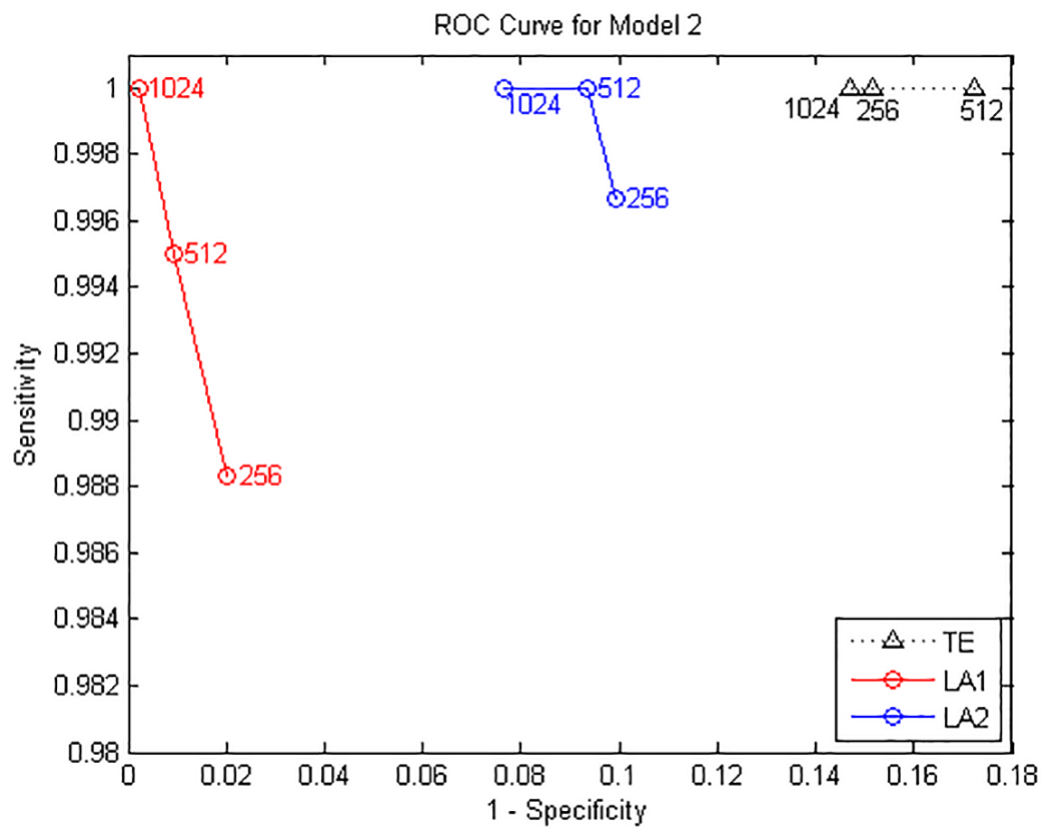
ROC curves for Model B. Sensitivity and specificity are obtained by gradually increasing the time series length from 256 to 1024 points.

The true causal connections in this model are at the matrix elements (1, 2), (1, 4), (2, 4), (4, 5), (5, 1) and (5, 2). The results from model B for data of 1024 points are shown in Fig 3. ROC curves are shown in Fig 4 and the values of sensitivity/specificity/F1 score listed in Table 2. The scales in Fig 4 are different from Fig 2 but the trends are similar: for the time series of 512/1024 points, both LA1 and LA2 give better performance than the original TE, and the specificity of LA1 is slightly higher than LA2 because the former is a more stringent criterion in the sense of the scaling factor. While for the traditional TE method, a large number of non-relevant connections were identified as significant, and the false positives distributed across the whole matrix, which also gave rise to a certain amount of redundant computation time. The outcome of Model A and B demonstrates that for linear systems the original TE from non-uniform embedding may bring about false positives at a rate higher than the nominal rate of *α* = 0.05, this fact has been already noticed by previous researchers [13], through introducing the low-dimensional approximation technique for the searching procedure in state space, our proposed method is able to avert this defect and retain the advantage of high sensitivity within the non-uniform embedding scheme.

**Table 2:** Sensitivity, specificity and F1 score values obtained from Model B.

	Sensitivity	Specificity	F1 score
256 points			
TE	1.000	0.849	0.850
LA1	0.988	0.980	0.971
LA2	0.997	0.901	0.895
512 points			
TE	1.000	0.828	0.833
LA1	0.995	0.991	0.987
LA2	1.000	0.906	0.902
1024 points			
TE	1.000	0.853	0.853
LA1	1.000	0.998	0.998
LA2	1.000	0.924	0.918

### Model C

Now we will consider three nonlinear models in which the binning and nearest neighbor estimator will be applied to evaluate the entropy. The first nonlinear model is composed by four time series which has the same coupling strength and causal direction as in the model A:

$$\begin{array}{c} \left\{ \begin{array}{ccc} x_{1}\left(t\right) & = & 0.8x_{1}\left(t-1\right)+0.65x_{2}\left(t-4\right)+\epsilon_{1}\left(t\right)\\ x_{2}\left(t\right) & = & 0.6x_{2}\left(t-1\right)+0.6x_{4}^{2}\left(t-5\right)+\epsilon_{2}\left(t\right)\\ x_{3}\left(t\right) & = & 0.5x_{3}\left(t-3\right)-0.6x_{1}^{2}\left(t-1\right)+0.4x_{2}\left(t-4\right)+\epsilon_{3}\left(t\right)\\ x_{4}\left(t\right) & = & 1.2x_{4}\left(t-1\right)-0.7x_{4}\left(t-2\right)+\epsilon_{4}\left(t\right) \end{array}\right. \end{array}$$ (11)

**Fig 5:**
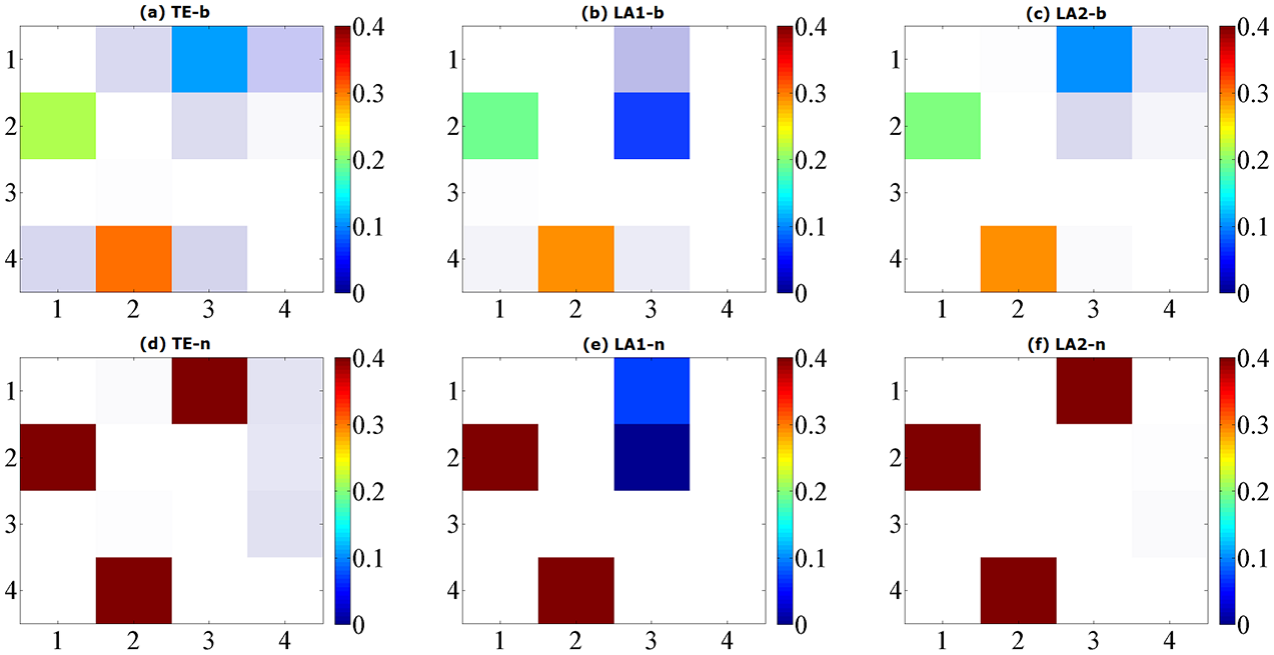
Matrix representations of the corresponding causalities for Model C. Retrieved by traditional TE method and two low-dimensional approximation methods, respectively by using binning entropy estimator (a, b, c) and NN entropy estimator (d, e, f) over 100 simulations of 1024 time points. The results are shown with color revealing the magnitude and transparency indicating the significance. The directions of causal influence are from row to column.

**Fig 6:**
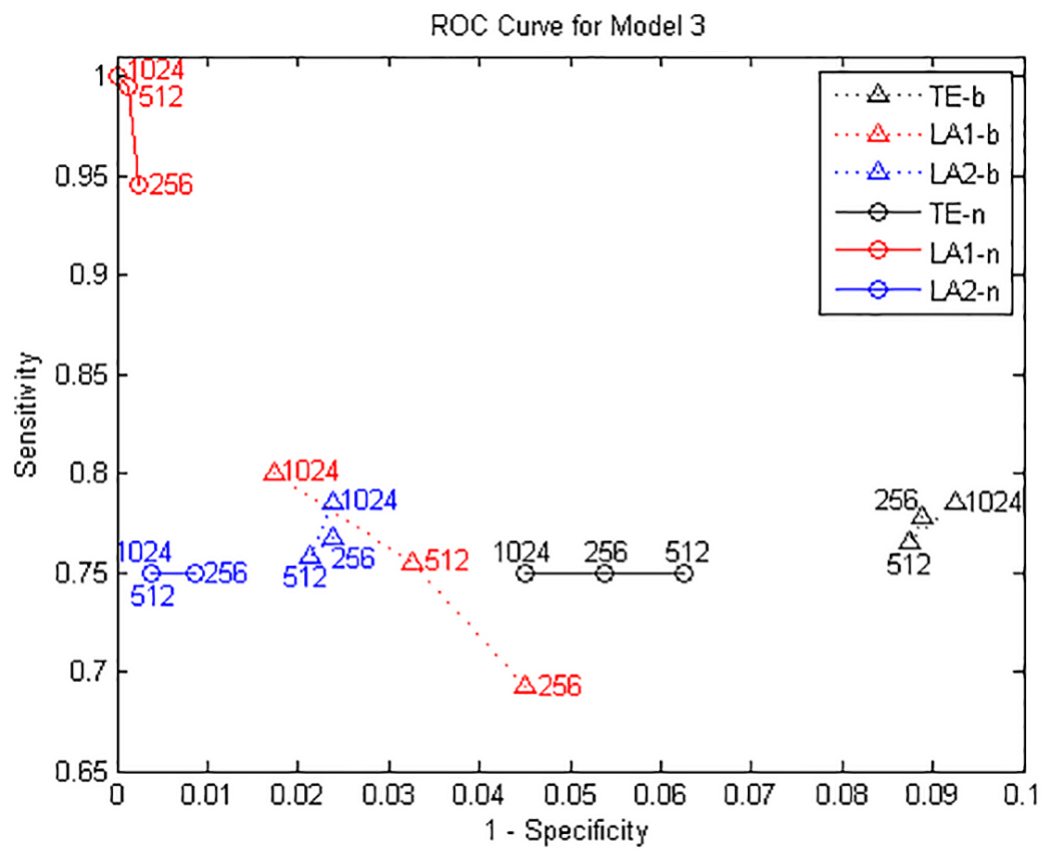
ROC curves for Model C. TE-b/LA1-b/LA2-b are the results by binning entropy estimator and TE-n/LA1-n/LA2-n by NN entropy estimator, sensitivity and specificity are obtained by gradually increasing the time series length from 256 to 1024 points.

The differences between model A and C are the nonlinear interactions in model C at the *X*_1_ → *X*_3_ and *X*_4_ → *X*_2_. For the calculation method of entropy we set 6 quantization levels for binning estimator and 10 nearest neighbors for NN estimator. The results for data of 1024 are shown in Fig 5, ROC curves shown in Fig 6 and the values of sensitivity/specificity/F1 score listed in Table 3, respectively labeled by the three computation methods (TE/LA1/LA2) and two entropy estimators (b/n). For the binning estimator, the specificity of LA1-b and LA2-b is higher than TE-b, with a comparably similar sensitivity. In the case of the NN estimator, traditional TE-n method failed to detect the causal relationship *X*_2_ → *X*_3_ for any data length, rendered a sensitivity of 75% at most. LA2-n also provided the same sensitivity with a slightly higher specificity, however LA1-n has successfully detected all the correct causal links in this example and attained the best sensitivity (100%) for 1024 points, though the value of TE at the *X*_1_ → *X*_3_ is relatively lower than the other methods.

**Table 3:** Sensitivity, specificity and F1 score values obtained from Model C by binning(-b) and NN(-n) estimators.

	Sensitivity	Specificity	F1 score
256 points			
TE-b	0.778	0.911	0.795
LA1-b	0.693	0.955	0.777
LA2-b	0.768	0.976	0.846
512 points			
TE-b	0.765	0.913	0.789
LA1-b	0.755	0.968	0.830
LA2-b	0.758	0.979	0.842
1024 points			
TE-b	0.785	0.908	0.797
LA1-b	0.800	0.983	0.872
LA2-b	0.785	0.976	0.857
256 points			
TE-n	0.750	0.946	0.808
LA1-n	0.945	0.998	0.969
LA2-n	0.750	0.991	0.849
512 points			
TE-n	0.750	0.938	0.800
LA1-n	0.995	0.999	0.996
LA2-n	0.750	0.996	0.853
1024 points			
TE-n	0.750	0.955	0.815
LA1-n	1.000	1.000	1.000
LA2-n	0.750	0.996	0.853

### Model D

The fourth example is another nonlinear VAR process of order 3 in 5 variables (the nonlinear model in [23], Eq (14)):

$$\begin{array}{c} \left\{ \begin{array}{ccc} x_{1}\left(t\right) & = & 0.95\sqrt{2}x_{1}\left(t-1\right)-0.9025x_{1}\left(t-2\right)+\epsilon_{1}\left(t\right)\\ x_{2}\left(t\right) & = & 0.5x_{1}^{2}\left(t-2\right)+\epsilon_{2}\left(t\right)\\ x_{3}\left(t\right) & = & -0.4x_{1}\left(t-3\right)+\epsilon_{3}\left(t\right)\\ x_{4}\left(t\right) & = & -0.5x_{1}^{2}\left(t-2\right)+0.25\sqrt{2}x_{4}\left(t-1\right)+0.25\sqrt{2}x_{5}\left(t-1\right)+\epsilon_{4}\left(t\right)\\ x_{5}\left(t\right) & = & -0.25\sqrt{2}x_{4}\left(t-1\right)+0.25\sqrt{2}x_{5}\left(t-1\right)+\epsilon_{5}\left(t\right) \end{array}\right. \end{array}$$ (12)

**Fig 7:**
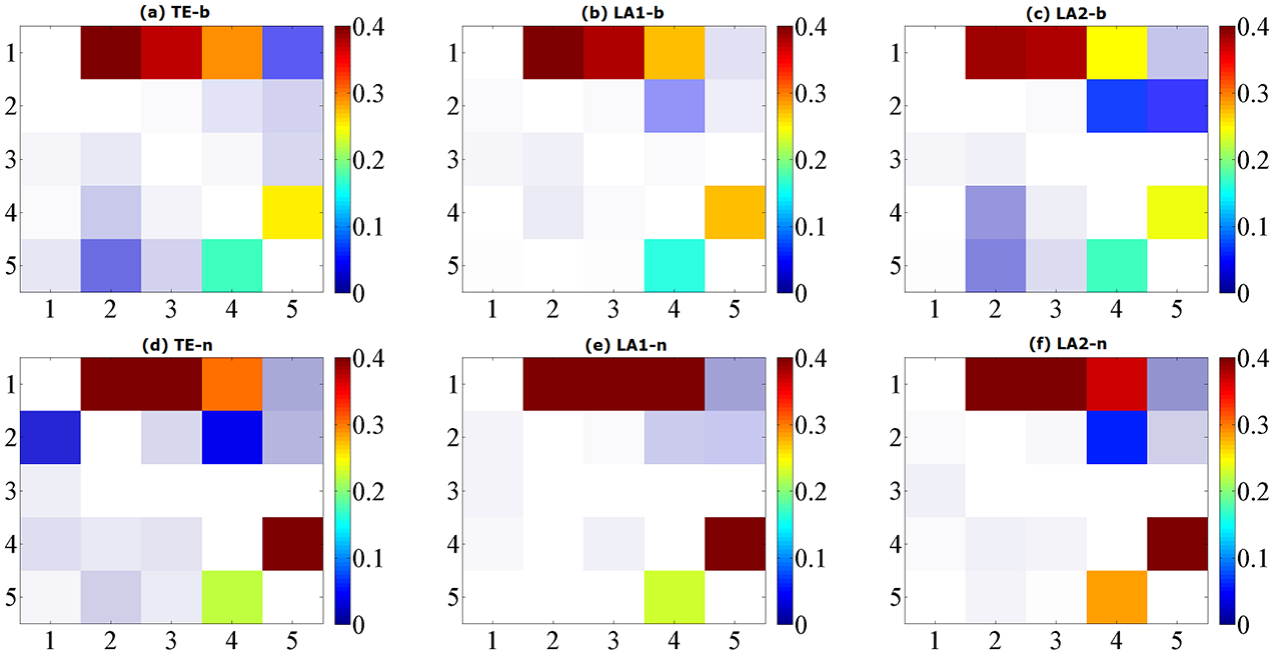
Matrix representations of the corresponding causalities for Model D. Retrieved by traditional TE method and two low-dimensional approximation methods, respectively by using binning entropy estimator (a, b, c) and NN entropy estimator (d, e, f) over 100 simulations of 1024 time points. The results are shown with color revealing the magnitude and transparency indicating the significance. The directions of causal influence are from row to column.

**Fig 8:**
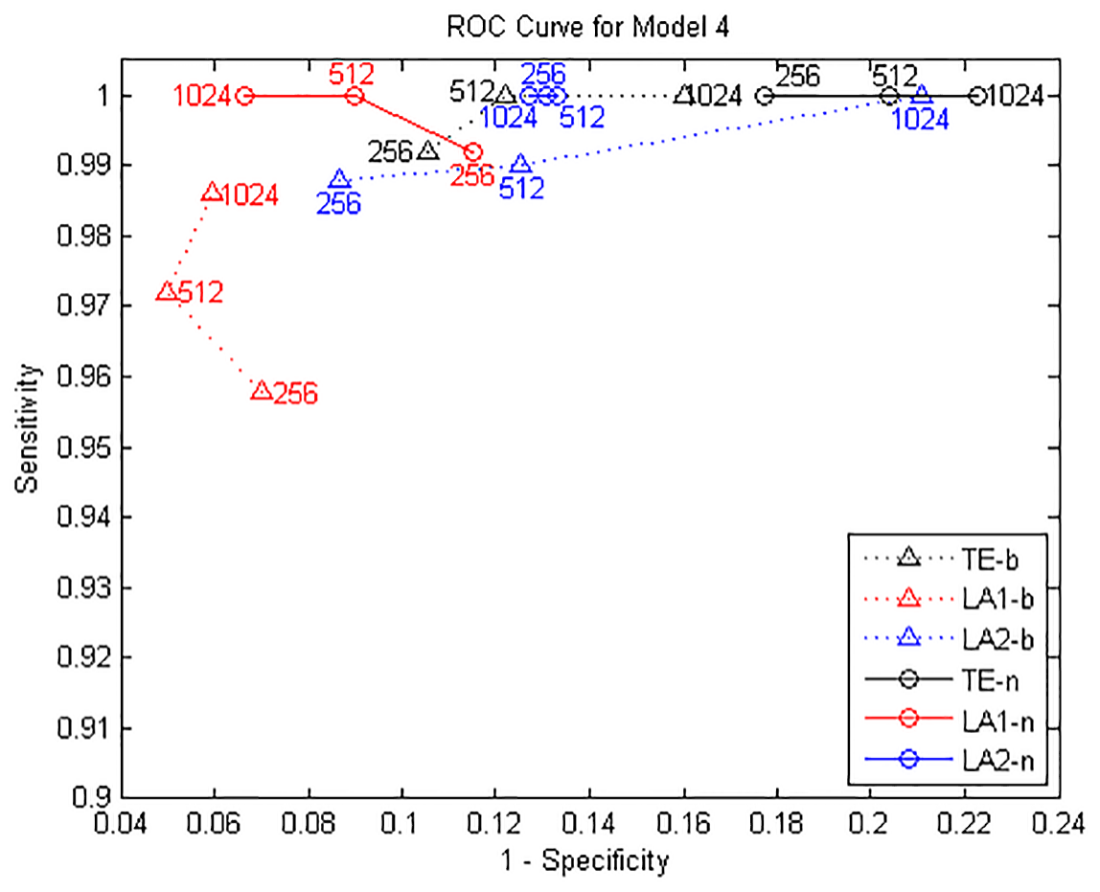
ROC curves for Model D. TE-b/LA1-b/LA2-b are the results by binning entropy estimator and TE-n/LA1-n/LA2-n by NN entropy estimator, sensitivity and specificity are obtained by gradually increasing the time series length from 256 to 1024 points.

The true causal connections in this model are at the matrix elements (1, 2), (1, 3), (1, 4), (4, 5), and (5, 4). The results for data of 1024 are shown in Fig 7, ROC curves shown in Fig 8 and the values of sensitivity/specificity/F1 score listed in Table 4. For the binning estimator, LA2-b presented a lower specificity compared LA1-b, but it was able to retrieve all the true links for 1024 points. The sensitivity of LA1-b is a bit lower than 1, which is improved by LA1-n. Regarding the TE method, the outcome of TE-n is worse than TE-b, despite the fact that the nearest neighbor estimator as a locally adaptive kernel estimator is more efficient for calculating entropies in high-dimensional spaces for limited data. In contrast, the proposed low-dimensional approximation technique for TE exploits the advantage of nearest neighbor estimator and attains better performance for both methods under the data length of 1024, in which the detection accuracy for the causal drivers is 100%.

**Table 4:** Sensitivity, specificity and F1 score values obtained from Model D by binning(-b) and NN(-n) estimators.

	Sensitivity	Specificity	F1 score
256 points			
TE-b	0.992	0.895	0.860
LA1-b	0.958	0.930	0.884
LA2-b	0.988	0.913	0.879
512 points			
TE-b	1.000	0.878	0.845
LA1-b	0.972	0.950	0.916
LA2-b	0.990	0.875	0.837
1024 points			
TE-b	1.000	0.840	0.806
LA1-b	0.986	0.941	0.911
LA2-b	1.000	0.789	0.760
256 points			
TE-n	1.000	0.823	0.790
LA1-n	0.992	0.885	0.849
LA2-n	1.000	0.869	0.836
512 points			
TE-n	1.000	0.796	0.766
LA1-n	1.000	0.910	0.881
LA2-n	1.000	0.867	0.833
1024 points			
TE-n	1.000	0.777	0.750
LA1-n	1.000	0.933	0.909
LA2-n	1.000	0.873	0.840

### Model E

**Fig 9:**
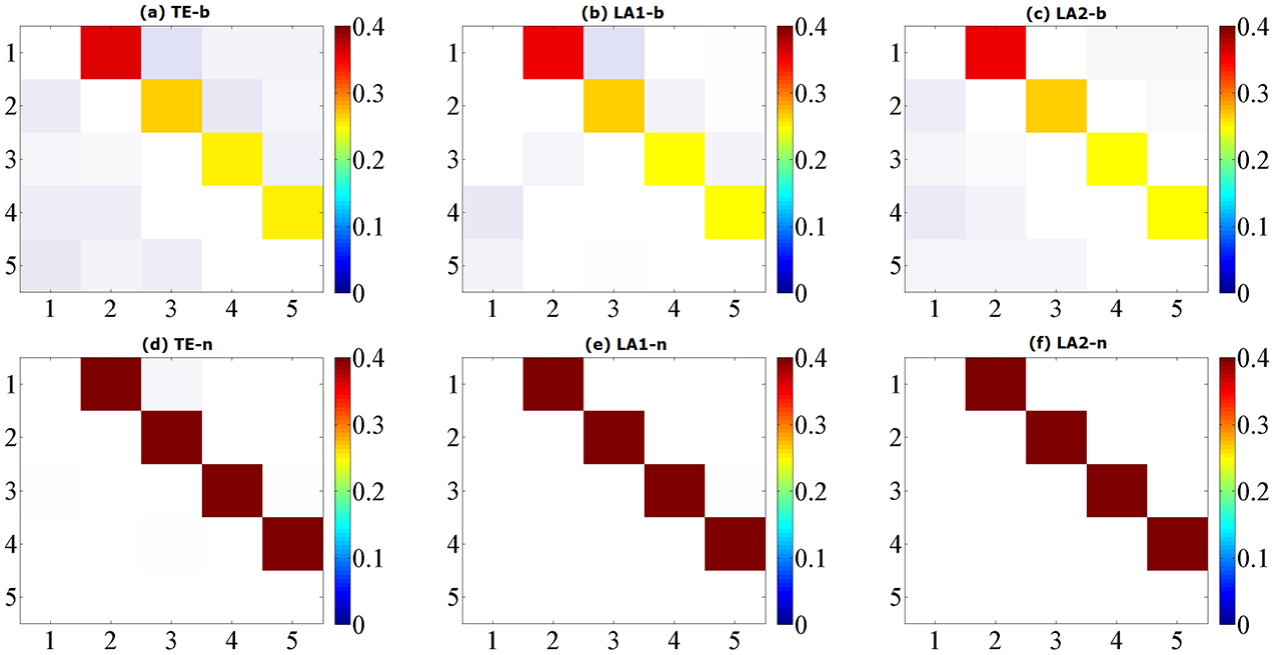
Matrix representations of the corresponding causalities for Model E. Retrieved by traditional TE method and two low-dimensional approximation methods, respectively by using binning entropy estimator (a, b, c) and NN entropy estimator (d, e, f) over 100 simulations of 1024 time points. The results are shown with color revealing the magnitude and transparency indicating the significance. The directions of causal influence are from row to column.

**Fig 10:**
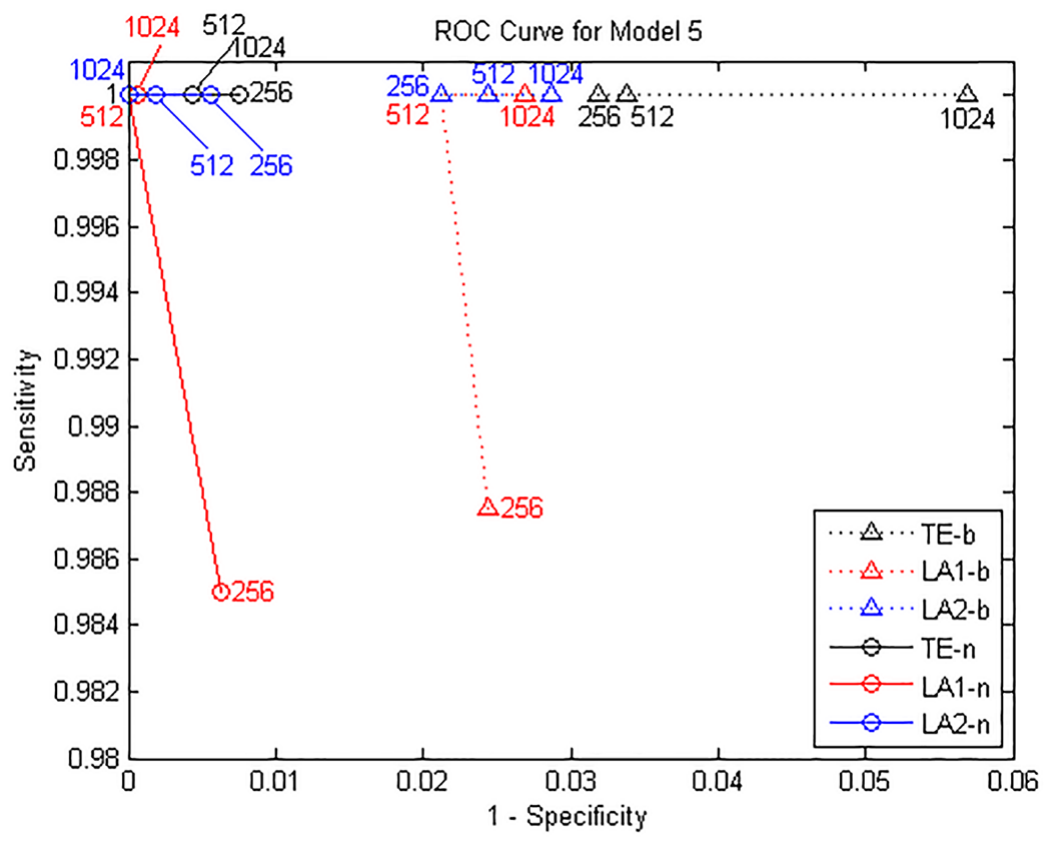
ROC curves for Model E. TE-b/LA1-b/LA2-b are the results by binning entropy estimator and TE-n/LA1-n/LA2-n by NN entropy estimator, sensitivity and specificity are obtained by gradually increasing the time series length from 256 to 1024 points.

In the fifth example we consider a lattice of five unidirectionally coupled noisy logistic maps,

$$\begin{array}{c} \left\{ \begin{array}{ccc} x_{1}\left(t\right) & = & \left(1-1.8x_{1}^{2}\left(t-1\right)\right)+0.01\epsilon_{1}\left(t\right)\\ x_{i}\left(t\right) & = & \left(1-\rho \right)\left(1-1.8x_{i}^{2}\left(t-1\right)\right)+\rho \left(1-1.8x_{i-1}^{2}\left(t-1\right)\right)+0.01\epsilon_{i}\left(t\right)\;i=2,\ldots ,5 \end{array}\right. \end{array}$$ (13)

where *ρ* = 0.2 is the coupling strength and *ϵ* are unit variance Gaussian noise terms. In this system, the first variable is evolving autonomously, whereas the other variables are influenced by the previous one with coupling *ρ*, thus forming a cascade of interactions *x*_*i*−1_ → *x*_*i*_. The results for data of 1024 are shown in Fig 9, ROC curves shown in Fig 10 and the values of sensitivity/specificity/F1 score listed in Table 5. Since the causal relationships in this model are relatively regular, all the three methods are able to identify the true interaction links and provide a good sensitivity and specificity, and the results from NN entropy estimator are all better than the binning estimator for this model. For the longest data of 1024 points the best result is given by LA2-n, which is nearly the same as LA1-n. It shows that with a proper data size, the F1 score obtained by LA1 and LA2 are always higher than the TE method.

**Table 5:** Sensitivity, specificity and F1 score values obtained from Model E by binning(-b) and NN(-n) estimators.

	Sensitivity	Specificity	F1 score
256 points			
TE-b	1.000	0.968	0.940
LA1-b	0.988	0.976	0.947
LA2-b	1.000	0.979	0.959
512 points			
TE-b	1.000	0.966	0.937
LA1-b	1.000	0.979	0.959
LA2-b	1.000	0.976	0.954
1024 points			
TE-b	1.000	0.943	0.898
LA1-b	1.000	0.973	0.949
LA2-b	1.000	0.971	0.946
256 points			
TE-n	1.000	0.993	0.985
LA1-n	0.985	0.994	0.980
LA2-n	1.000	0.994	0.989
512 points			
TE-n	1.000	0.996	0.991
LA1-n	1.000	1.000	1.000
LA2-n	1.000	0.998	0.996
1024 points			
TE-n	1.000	0.996	0.991
LA1-n	1.000	0.999	0.999
LA2-n	1.000	1.000	1.000

**Fig 11:**
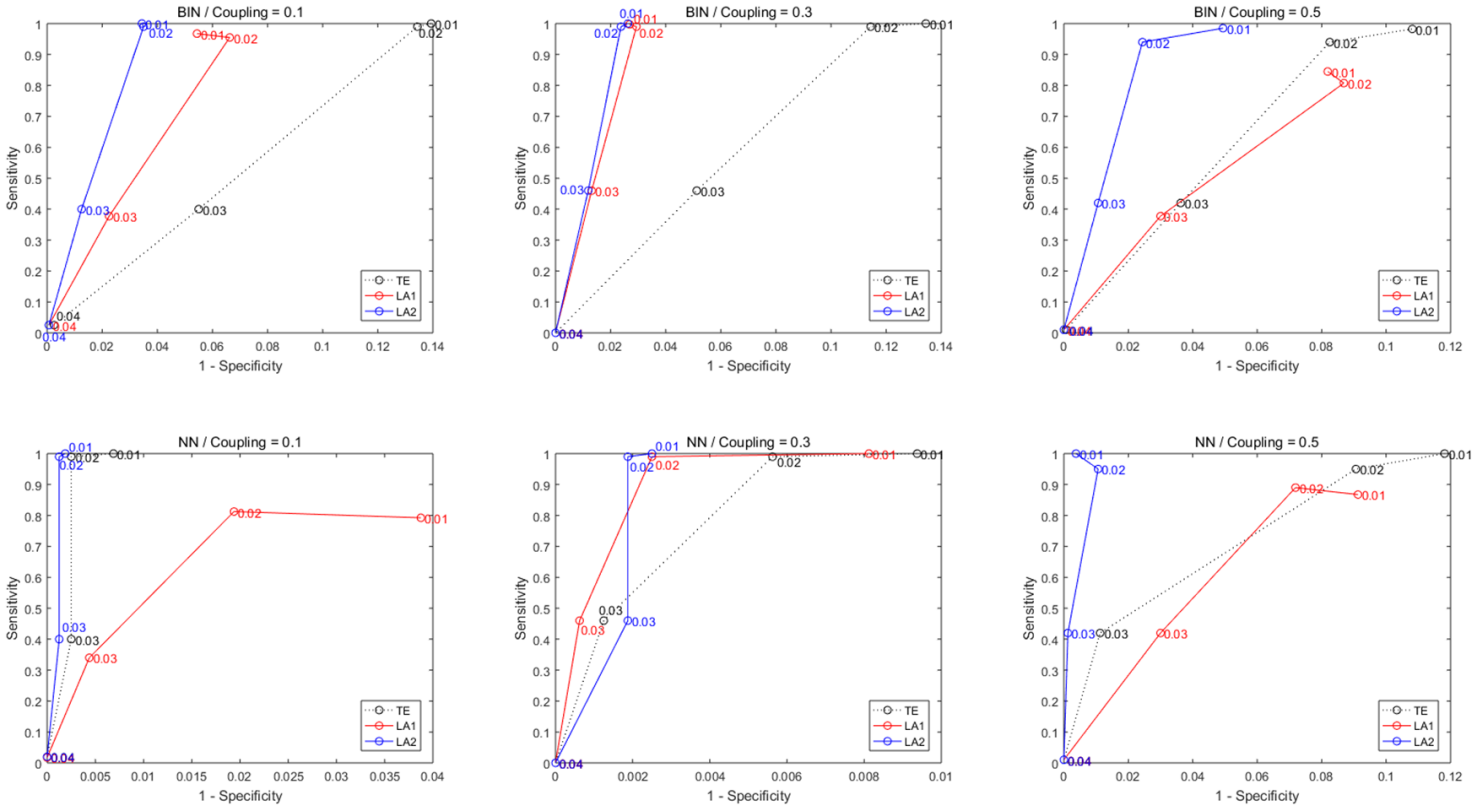
ROC curves for Model E with varying noise and coupling strengths. Sensitivity and specificity are obtained for models with a varying noise from 0.01 to 0.04 (marked in the figure). The first row shows results from the methods applying binning estimator and second row for NN estimator. Column 1 to 3 is with different coupling strength from 0.1, 0.3 to 0.5. Each simulation was performed 100 runs with data length 512.

In this example the noise term is defined by a superimposed form with a relatively little strength in accordance with previous researches [35, 36]. When noise strength is larger than 0.04 all the methods failed to detect causal relationships between the variables. To illustrate this, we conducted an experiment varying the noise strength from 0.01 to 0.04 and the coupling strength *ρ* = 0.1, 0.3, 0.5, and depicted the results in Fig 11 (data length = 512). In the results by using binning estimator, it shows that the both LA methods perform better than original TE method. Except in the example of weak coupling by NN estimator, the method of LA1 gave out a relative low sensitivity, while the LA2 is still superior to the traditional TE.

### Model F

Next we consider three coupled Henon maps

$$\begin{array}{c} \left\{ \begin{array}{ccc} x_{1}\left(t\right) & = & 1.4-x_{1}^{2}\left(t-1\right)+0.3x_{1}\left(t-2\right)\\ x_{2}\left(t\right) & = & 1.4-cx_{1}\left(t-1\right)x_{2}\left(t-1\right)-\left(1-c\right)x_{2}^{2}\left(t-1\right)+0.3x_{2}\left(t-2\right)\\ x_{3}\left(t\right) & = & 1.4-cx_{2}\left(t-1\right)x_{3}\left(t-1\right)-\left(1-c\right)x_{3}^{2}\left(t-1\right)+0.3x_{3}\left(t-2\right) \end{array}\right. \end{array}$$ (14)

**Fig 12:**
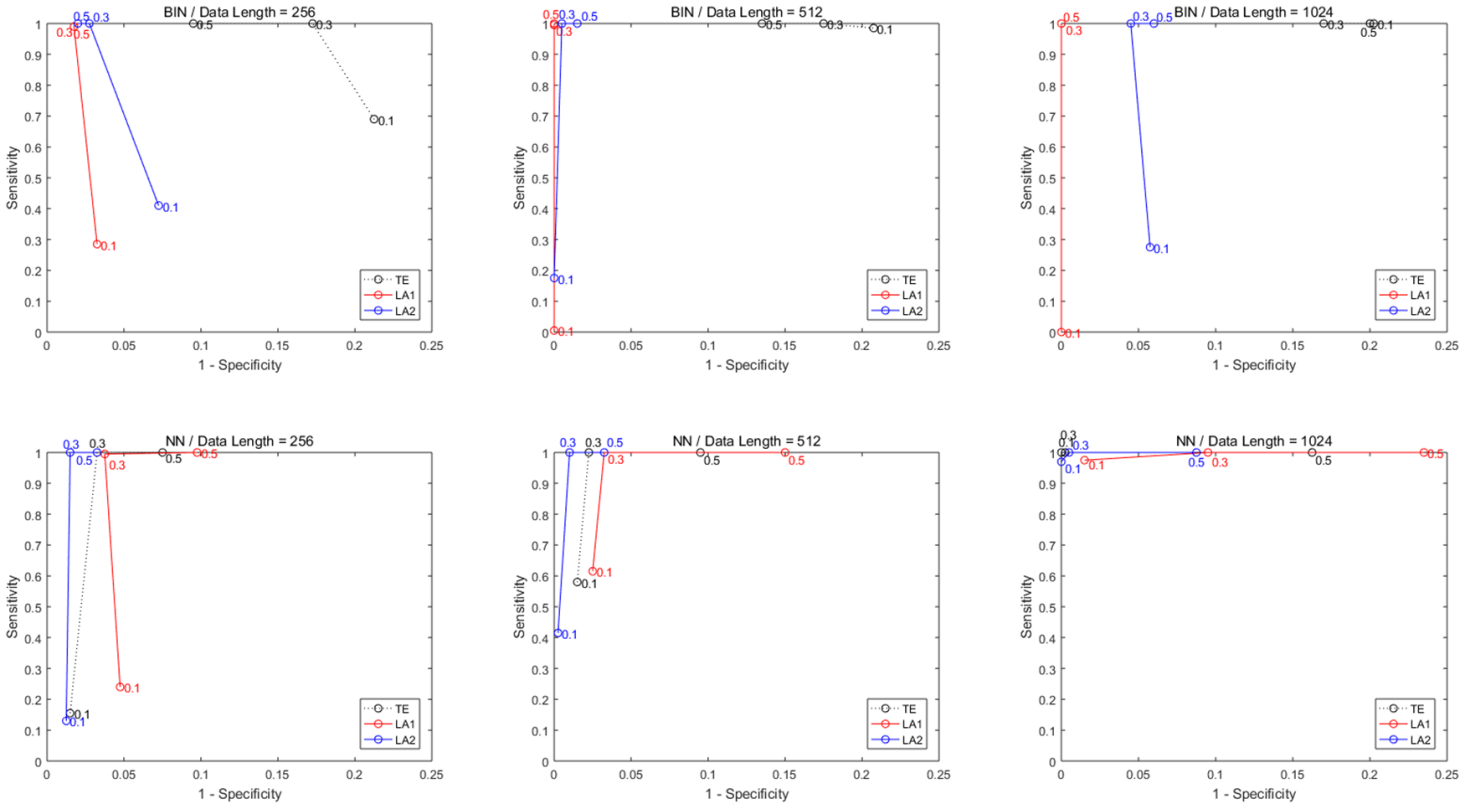
ROC curves for Model F with different data lengths and coupling strengths. Sensitivity and specificity are obtained from three coupled Henon maps with data length from 256 to 1024 and coupling strength (marked in the figure) from 0.1 to 0.5. The first row shows results from the methods applying binning estimator and second row for NN estimator. Column 1 to 3 is with data length from 256 to 1024. Each simulation was performed by 100 runs.

Nonlinear couplings *x*_1_ → *x*_2_ and *x*_2_ → *x*_3_ are defined with equal coupling strengths c = 0.1, 0.3, 0.5, under which the system does not become completely synchronized. The results of ROC curves for different data lengths and coupling strengths are shown in Fig 12, and the values of sensitivity/specificity/F1 score listed in Table 6. In the case of weak coupling (c = 0.1), both LA methods could not achieve a good sensitivity compared to traditional TE (except for the 1024 data by NN estimator). But for the data of medium and strong coupling (c = 0.3, 0.5), the sensitivity of LA methods reached 100% together with a high specificity. In results by binning estimator, the LA1 method performs better than the other two, especially for data of longer length (512, 1024), while by NN estimator LA2 is more advantageous. Moreover, the specificity of all three methods by NN estimator tends to decrease with the coupling strength of model.

**Table 6:** Sensitivity, specificity and F1 score values obtained from Model F by binning(-b) and NN(-n) estimators with different data lengths and coupling strengths.

Coupling	Sen	Spe	F1	Sen	Spe	F1	Sen	Spe	F1
	TE-b/256			LA1-b/256			LA2-b/256		
0.1	0.690	0.213	0.652	0.285	0.033	0.422	0.410	0.073	0.527
0.3	1.000	0.173	0.853	0.990	0.018	0.978	1.000	0.028	0.973
0.5	1.000	0.095	0.913	1.000	0.020	0.980	1.000	0.020	0.980
	TE-b/512			LA1-b/512			LA2-b/512		
0.1	0.985	0.208	0.821	0.005	0.000	0.010	0.175	0.000	0.298
0.3	1.000	0.175	0.851	0.995	0.000	0.997	1.000	0.005	0.995
0.5	1.000	0.135	0.881	1.000	0.000	1.000	1.000	0.015	0.985
	TE-b/1024			LA1-b/1024			LA2-b/1024		
0.1	1.000	0.203	0.832	0.000	0.000	0.000	0.275	0.058	0.396
0.3	1.000	0.170	0.855	1.000	0.000	1.000	1.000	0.045	0.957
0.5	1.000	0.200	0.833	1.000	0.000	1.000	1.000	0.060	0.943
	TEn/256			LA1n/256			LA2n/256		
0.1	0.155	0.015	0.262	0.240	0.048	0.360	0.130	0.013	0.225
0.3	1.000	0.033	0.969	0.995	0.038	0.961	1.000	0.015	0.985
0.5	1.000	0.075	0.930	1.000	0.098	0.911	1.000	0.033	0.969
	TEn/512			LA1n/512			LA2n/512		
0.1	0.580	0.015	0.720	0.615	0.025	0.739	0.415	0.002	0.585
0.3	1.000	0.023	0.978	1.000	0.033	0.969	1.000	0.010	0.990
0.5	1.000	0.095	0.913	1.000	0.150	0.870	1.000	0.033	0.969
	TEn/1024			LA1n/1024			LA2n/1024		
0.1	1.000	0.000	1.000	0.975	0.015	0.973	0.970	0.000	0.985
0.3	1.000	0.002	0.998	1.000	0.095	0.913	1.000	0.005	0.995
0.5	1.000	0.163	0.860	1.000	0.235	0.810	1.000	0.088	0.920

### Model G

**Fig 13:**
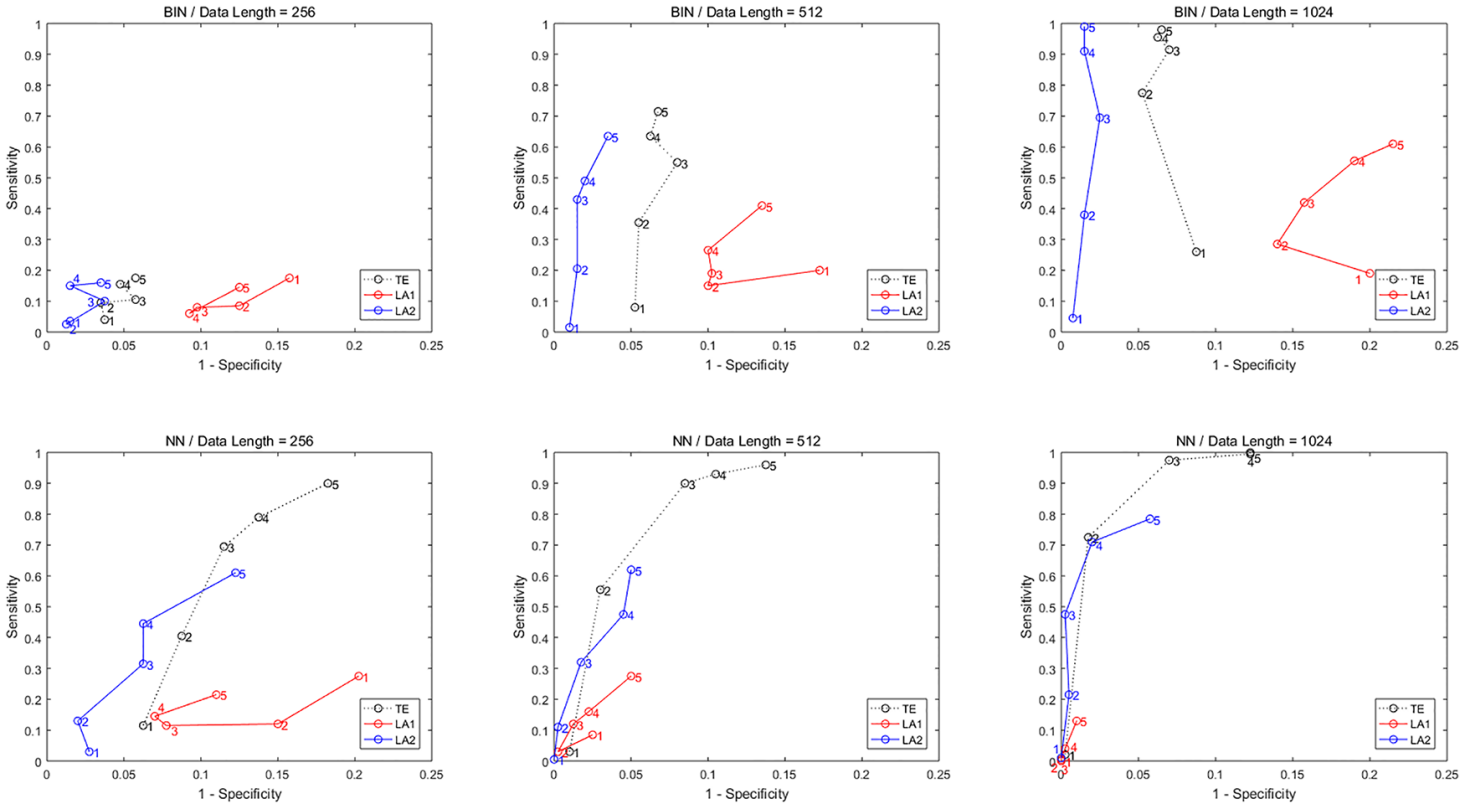
ROC curves for Model G with different data lengths and coupling strengths. Sensitivity and specificity are obtained from three coupled Lorenz oscillators with data length from 256 to 1024 and coupling strength (marked in the figure) from 1 to 5. The first row shows results from the methods applying binning estimator and second row for NN estimator. Column 1 to 3 is with data length from 256 to 1024. Each simulation was performed by 100 runs.

In the last simulation, a system of three coupled identical Lorenz oscillators is defined as

$$\begin{array}{c} \left\{ \begin{array}{cc} {\dot{x}}_{1} & =10(y_{1}-x_{1})\\ {\dot{y}}_{1} & =28x_{1}-y_{1}-x_{1}z_{1}\\ {\dot{z}}_{1} & =x_{1}y_{1}-\frac{8}{3}z_{1} \end{array}\begin{array}{cc} {\dot{x}}_{2} & =10\left(y_{2}-x_{2}\right)+c\left(x_{1}-x_{2}\right)\\ {\dot{y}}_{2} & =28x_{2}-y_{2}-x_{2}z_{2}\\ {\dot{z}}_{2} & =x_{2}y_{2}-\frac{8}{3}z_{2} \end{array}\begin{array}{cc} {\dot{x}}_{3} & =10\left(y_{3}-x_{3}\right)+c\left(x_{2}-x_{3}\right)\\ {\dot{y}}_{3} & =28x_{3}-y_{3}-x_{3}z_{3}\\ {\dot{z}}_{3} & =x_{3}y_{3}-\frac{8}{3}z_{3} \end{array}\right. \end{array}$$ (15)

with couplings *x*_1_ → *x*_2_ and *x*_2_ → *x*_3_ of equal strengths c = 1, 2, 3, 4, 5. The first variables *x*_*i*_ of the three interacting subsystems are respectively observed at a sampling time of 0.05 units, and the differential equations is solved using the explicit Runge-Kutta (4, 5) method “ode45” in MATLAB. The results of ROC curves for different data lengths and coupling strengths are shown in Fig 13, and the values of sensitivity/specificity/F1 score listed in Table 7. In most cases of this example, the traditional TE method outperformed LA methods, which indicates that this coupled continuous chaotic system cannot benefit from low-dimensional approximation strategy. The results for LA1 method are especially worse than LA2, partly due to the inclusion/exclusion of the higher order interactions among variables. However in the simulation of larger coupling strength (c = 4, 5) with data length of 1024 by binning method, LA2 achieves higher F1 score than TE, and with longer data the performance of LA2 by NN estimator is also significantly improved. This fact demonstrates that for LA2 method, data size is crucial for the estimation of higher-dimensional influences.

**Table 7:** Sensitivity, specificity and F1 score values obtained from Model G by binning(-b) and NN(-n) estimators with different data lengths and coupling strengths.

Coupling	Sen	Spe	F1	Sen	Spe	F1	Sen	Spe	F1
	TE-b/256			LA1-b/256			LA2-b/256		
1	0.040	0.038	0.072	0.175	0.158	0.235	0.035	0.015	0.066
2	0.095	0.035	0.163	0.085	0.125	0.127	0.025	0.013	0.048
3	0.105	0.058	0.172	0.080	0.098	0.125	0.100	0.038	0.170
4	0.155	0.048	0.248	0.060	0.093	0.096	0.150	0.015	0.254
5	0.175	0.058	0.271	0.145	0.125	0.208	0.160	0.035	0.260
	TE-b/512			LA1-b/512			LA2-b/512		
1	0.080	0.053	0.135	0.200	0.173	0.259	0.015	0.010	0.029
2	0.355	0.055	0.485	0.150	0.100	0.222	0.205	0.015	0.332
3	0.550	0.080	0.643	0.190	0.103	0.272	0.430	0.015	0.589
4	0.635	0.063	0.722	0.265	0.100	0.362	0.490	0.020	0.641
5	0.715	0.068	0.773	0.410	0.135	0.488	0.635	0.035	0.745
	TE-b/1024			LA1-b/1024			LA2-b/1024		
1	0.260	0.088	0.362	0.190	0.200	0.239	0.045	0.007	0.085
2	0.775	0.053	0.824	0.285	0.140	0.364	0.380	0.015	0.539
3	0.915	0.070	0.891	0.420	0.158	0.484	0.695	0.025	0.797
4	0.955	0.063	0.918	0.555	0.190	0.574	0.910	0.015	0.938
5	0.980	0.065	0.929	0.610	0.215	0.598	0.990	0.015	0.980
	TE-n/256			LA1-n/256			LA2-n/256		
1	0.115	0.063	0.185	0.275	0.203	0.327	0.030	0.028	0.055
2	0.405	0.088	0.513	0.120	0.150	0.169	0.130	0.020	0.222
3	0.695	0.115	0.722	0.115	0.078	0.181	0.315	0.063	0.438
4	0.790	0.138	0.765	0.145	0.070	0.226	0.445	0.063	0.567
5	0.900	0.183	0.795	0.215	0.110	0.300	0.610	0.123	0.658
	TE-n/512			LA1-n/512			LA2-n/512		
1	0.030	0.010	0.057	0.085	0.025	0.150	0.005	0.000	0.010
2	0.555	0.030	0.687	0.030	0.002	0.058	0.110	0.002	0.197
3	0.900	0.085	0.870	0.120	0.013	0.210	0.320	0.018	0.472
4	0.930	0.105	0.869	0.160	0.023	0.266	0.475	0.045	0.607
5	0.960	0.138	0.859	0.275	0.050	0.400	0.620	0.050	0.721
	TE-n/1024			LA1-n/1024			LA2-n/1024		
1	0.020	0.002	0.039	0.000	0.000	0.000	0.010	0.000	0.020
2	0.725	0.018	0.824	0.005	0.000	0.010	0.215	0.005	0.351
3	0.975	0.070	0.922	0.010	0.000	0.020	0.475	0.002	0.642
4	0.995	0.123	0.888	0.040	0.002	0.077	0.710	0.020	0.811
5	1.000	0.123	0.891	0.130	0.010	0.226	0.785	0.058	0.826

## Application

**Fig 14:**
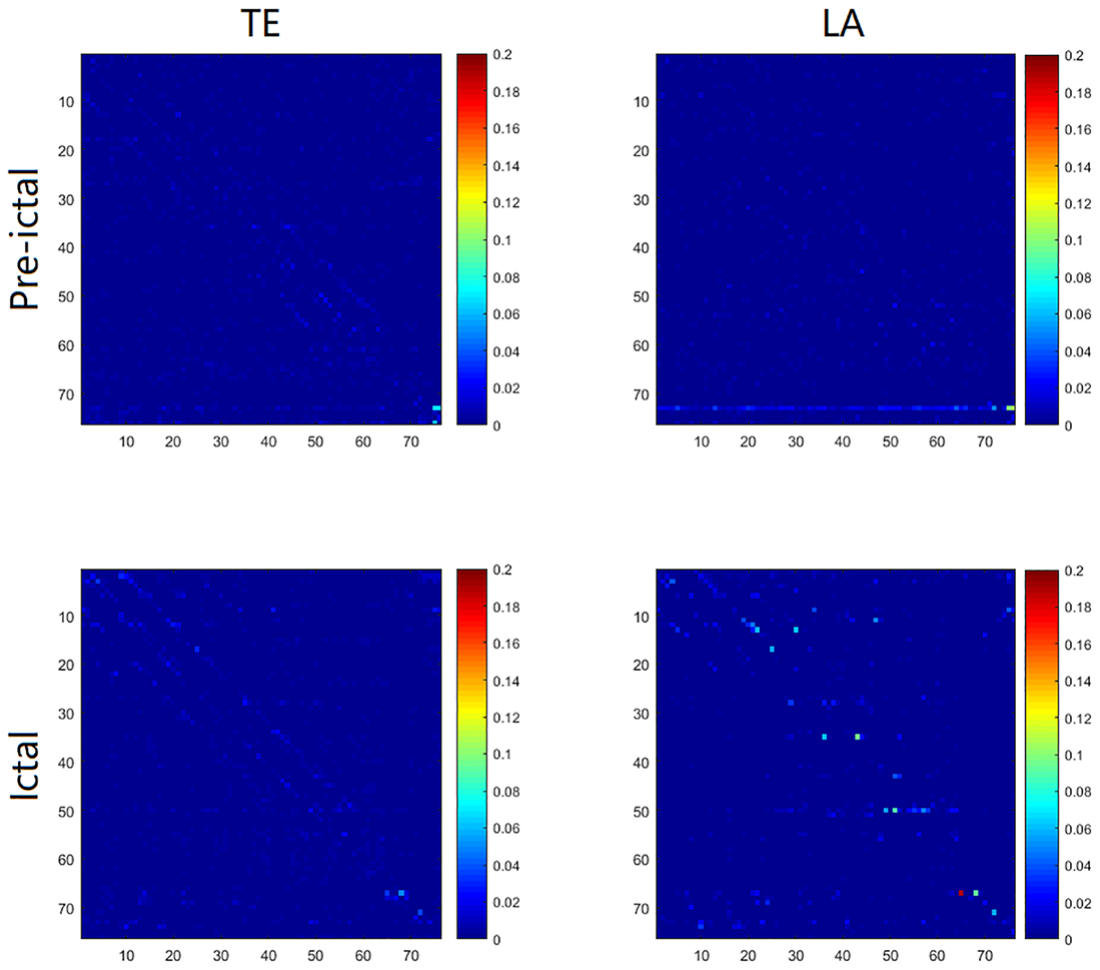
Results for an epileptic EEG recording. Matrices of causalities reflect before (top) and during (down) the clinical onset of a seizure from an epileptic patient, the results are respectively averaged from 8 recordings. Contacts 1 to 64 belong to a cortical electrode grid, and contacts 65 to 76 to two depth electrode strips. The values are computed by traditional non-uniform transfer entropy (TE) and low-dimensional approximation approach (LA). The directions of causal influence are from row to column. The brighter colors correspond to more significant values.

**Fig 15:**
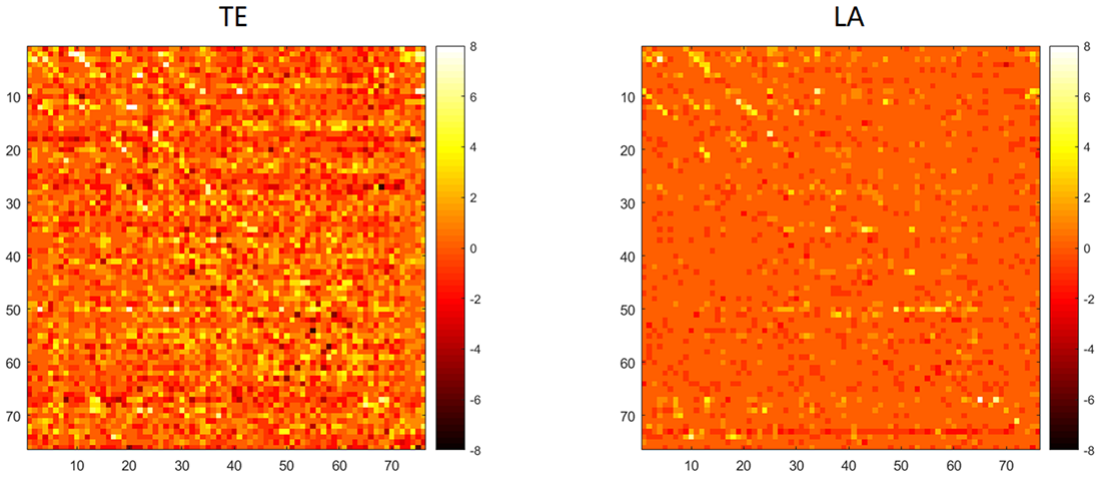
Results for an epileptic EEG recording. Matrices depict the difference of total numbers of significant connections between ictal and pre-ictal period (ict—pre). The numbers are respectively summed from 8 recordings. Contacts 1 to 64 belong to a cortical electrode grid, and contacts 65 to 76 to two depth electrode strips. The values are computed by traditional non-uniform transfer entropy (TE) and low-dimensional approximation approach (LA). The directions of causal influence are from row to column. The brighter colors correspond to more significant values.

In this section we turn to real-world data to show the applicability of our proposed approach for non-uniform embedding. To address this issue we consider a public dataset [37] from an epileptic patient with implanted array of 8-by-8 cortical electrode grid and two depth electrode strips with six contacts each, which amount to 76 time series (More details about the data are given in [38]). For its intrinsic high dimensionality and redundancy, this data is intuitively appropriate to be employed a non-uniform embedding method to disentangle the underlying dynamical interactions. Therefore we applied our low-dimensional approximation method (LA1) to analyze the data corresponding to 8 epileptic seizures and 8 periods just before the seizure onset, respectively averaged the ictal/pre-ictal results and then compared its performance with traditional non-uniform embedding transfer entropy, both by the implementation of nearest neighbor estimator. In this application the data which were recorded at 400 Hz of 10 seconds length were downsampled to 100 Hz, and the maximum embedding order was set to 8. The results are depicted in Fig 14, which shows the matrices of causalities before and during the clinical onset of the seizure, and Fig 15, which shows the difference of total numbers of significant connections between ictal and pre-ictal period, respectively by TE and LA method. From matrix representation by the LA method for the pre-ictal data we note that an obvious causal driver is located at the contact 73 from the second depth electrode strip, this contact can thus be associated to the seizure onset and reflects the fact that the seizure is already active even if it is not yet clinically observable, also in [35, 39], it has been suggested that the last two contacts in the second depth electrode are mostly influencing the cortical activity. The results from ictal data indicates abnormal interactions from the regions corresponding to the lower left corner of the grid (contacts 1–4, 9–11 and 17) which were then resected during an anterior temporal lobectomy for the patient. Moreover, from the ictal period our method has successfully identified a critical node: contact 50, which exhibits the most significant change in the value of betweenness centrality and was considered as a target for therapeutic intervention in [38], for the reason that these nodes with statistically significant increases in betweenness centrality may facilitate seizure activity and their disruption could prevent or abort ictal activity. The traditional TE method, on the contrary, leads to more noisy results with a significant number of false positives, which has extended the computation time to about three folds larger, and it is more sensitive to the confounding effect of volume conduction resulting in the diagonal patterns observed in the matrix compared with our method.

## Discussion and conclusion

In this article we have presented a novel and effective modification for the well-known causality analysis tool: transfer entropy from non-uniform embedding, which is the state-of-the-art for quantifying causal networks by means of information theoretic measures and has been employed by a large number of researchers from a variety of disciplines over recent years. Besides the widely used transfer entropy as a model-free approach there are also other forms of causality measures that based on different criterion rather than the maximization of CMI and has shown its great statistical power in detecting relationships from a multivariate embedding space [40], e.g. local predictability (LP). For the computation of transfer entropy, the employment of non-uniform embedding strategy has been proved to be suitable for high dimensional embedding spaces because of its ability to reduce the effective dimension of the state space by selecting only the relevant variables at specific time lags that contribute the most to explain the target variable. Thus it is a more flexible procedure for the reconstruction of the multivariate embedding space compared to the uniform embedding and has been proposed by several previous authors [7, 22, 41]. Nevertheless it still encounters the obstacle of estimation for CMI among the candidate vectors and tends to detect false directed couplings. Our suggested approach exploits low-dimensional approximation method that derives from the feature selection framework to improve the termination criterion in the procedure of constructing embedding vectors. The detailed approximation strategies we used are formed in two different ways: one is a stringent version of JMI criterion and the other is RelaxMRMR criterion that takes into account the second order interactions. In a series of simulations we compared the performance of our algorithm with traditional TE implementation under various entropy estimators. We reported the sensitivity and specificity of the results from different data lengths, confirmed the capability of the proposed approach, which could lead to less false positives in the detection of causal flow while retaining the advantage of non-uniform embedding in terms of high sensitivity, especially for the large data sets.

The main development in this paper is that instead of directly applying original CMI as a criterion for identifying significant variables at specific lags as in recent literature, our approach follows a paradigm that makes use of low-dimensional mutual information quantities to approximate higher dependencies between candidate and target variables in the embedding vector reconstruction. It effectively avoids the curse of dimensionality and achieves a more parsimonious model by maximizing relevance and minimizing redundancy between the selected components. To tackle this kind of problem there also exists other propositions, e.g. in [42] the authors introduced a preselection scheme for the subsets of causal predictors to overcome the combinatorial explosion for searching a globally optimal subset and detect the synergetic variables, while for our method it relies on the low-dimensional approximation to alleviate this condition. In the simulation experiments, the approach LA1 with an outweighed factor on the redundancy and conditional redundancy term generally outperformed LA2 which took higher order interactions of embedding vectors at different lags into consideration, and presented the best possible results throughout most of the examples except for the coupled Lorenz system as long as data size is large enough for good statistic. The reason that LA2 did not show much obvious advantage compared to LA1 may partly due to the number of candidate variables in state space is limited to a small number with a small time delay, thus renders the underlying causal structure of the data set not complex enough. In this case a simple criterion such as LA1 may already be sufficiently capable to give a reasonable outcome. Another fact is that the performance of an approximation method is not only affected by the dimensionality of the state space, but also by the data size. Therefore a large data is crucial for the estimation of higher-dimensional influences, which has been shown in the last example.

As with the proposed methodology there are also some limitations. For instance, although in the simulations of linear model our algorithm produced a better result compared to the original non-uniform transfer entropy, it still could not surpass the efficiency of the standard uniform conditioning methods [23], and it is more computationally intensive for the associated randomization significance tests, which is intrinsically required under the non-uniform conditioning framework. For this reason the beneficial effects of applying the low-dimensional approximations for TE may rely mostly upon the nonlinear causal relationships, like in the EEG or MEG data. Also in the experiments of chaotic systems our method did not show obvious advantage compared to TE, and sometimes even failed to detect the underlying causal relationship, which restricted its range of applicability and this issue should be investigated in our further work. In [43], the authors had carried out a simulation study to compare the performance of several causality measures and concluded that the non-uniform TE leading to the best in the case of nonlinear simulation systems and always obtaining the highest specificity. In our simulations this was confirmed that the traditional TE method could already give a 100% sensitivity and nearly 0 false positives and leaves little space to be improved, this may to some extent explain what kind of system is most suitable for the applying of non-uniform scheme. Another issue for the low-dimensional approximation approach is that it is prone to bring about more false negatives, especially for small data sets. A polished version of this method should address this problem theoretically, perhaps using an adaptive balancing factor in which the focus may shift between the relevance and redundancy terms as the unexplained information of the target variable decreases from the earlier to the latter stage of searching and constructing embedding vectors, rather than a fixed parameter pattern for the relevance/redundancy/conditional redundancy in the embedding vector selection process. For different entropy estimators, the effect of low-dimensional approximations differs in the trade-off between the information gain and the nonexistent coupling rejection. A more flexible termination criterion will certainly be helpful to discover the true coupling direction and latency in multivariate causality analysis.

## Supporting information

S1 File Matlab code for LA method by BIN-NN estimator. (ZIP)
